# Evaluation of Arcuate Uterus in Infertility Using 3D Transvaginal Ultrasound: A Retrospective Cohort Study

**DOI:** 10.1002/rmb2.70009

**Published:** 2025-12-27

**Authors:** Tatsuya Yoshihara, Maki Ogi, Dai Miyashita, Kota Tanaka, Yuri Tada, Osamu Yoshino

**Affiliations:** ^1^ Department of Obstetrics and Gynecology University of Yamanashi Yamanashi Japan

**Keywords:** 3D transvaginal ultrasound, arcuate uterus, infertility, unexplained infertility, uterine anomaly

## Abstract

**Purpose:**

To determine the prevalence and clinical significance of arcuate uterus among infertile women using three‐dimensional transvaginal ultrasound (3D‐TVUS).

**Methods:**

This retrospective cohort included 327 women evaluated for infertility between January 2021 and May 2025. Arcuate uterus was defined as fundal indentation < 10 mm and angle ≥ 90°. The primary outcome was live birth, with subgroup analysis in assisted reproductive technology (ART) cases. Logistic regression was used to assess independent associations.

**Results:**

Arcuate uterus was found in 19% (62/327) of infertile women and was more frequent in unexplained infertility than in explained cases (32% vs. 14%; cOR 2.82, 95% CI 1.59–5.00, *p* = 0.001). Live birth rates did not differ significantly between arcuate and normal uterus, overall (17% vs. 14%; aOR 1.3, 95% CI 0.2–5.9) or in ART (65% vs. 63%; aOR 1.1, 95% CI 0.5–2.4). Reproducibility was excellent (*κ* = 0.94).

**Conclusions:**

Arcuate uterus was relatively common, especially in unexplained infertility, but did not independently affect live birth. 3D‐TVUS assessment may help identify subgroups within unexplained infertility, warranting validation in multicenter studies.

## 1 Introduction

Uterine morphological abnormalities are one of the major anatomical factors associated with infertility and pregnancy complications [1, 2, 3]. These anomalies are thought to result from defects in the fusion or resorption of the Müllerian ducts during fetal development [4, 5]. Their prevalence has been reported to be approximately 0.4%–10% among women in the general population and 8%–25% among women with infertility [1, 6]. Among these anomalies, the arcuate uterus is the mildest form, characterized by a slight concavity at the uterine fundus, and its clinical significance has long been a matter of debate [1, 2, 4, 7].

Traditionally, the arcuate uterus has often been regarded as an “anatomical variation within the normal range [8, 9, 10].” However, several studies have suggested a possible association between this anomaly and adverse pregnancy outcomes, including implantation failure, recurrent miscarriage, preterm birth, and fetal growth restriction [3, 11, 12, 13]. On the other hand, clear evidence supporting the arcuate uterus as a risk factor for infertility or miscarriage has not been established. Accordingly, in the revised 2021 ASRM classification, it is not recognized as a distinct pathological category [14].

This discrepancy is considered to be largely attributable to inconsistencies in evaluation methods and classification criteria [2, 8, 14, 15]. In recent years, the widespread use of three‐dimensional transvaginal ultrasound (3D‐TVUS) has enabled objective and highly reproducible evaluation of uterine morphology, leading to renewed efforts to redefine the arcuate uterus and reassess its clinical significance [3, 8].

The aim of this study was to clarify the prevalence of arcuate uterus among infertile women using objective morphological assessment with 3D‐TVUS. Furthermore, we sought to compare the clinical characteristics and association with infertility between women with arcuate and normal uterine morphology, and to evaluate the impact of arcuate uterus on live birth rates.

## 2 Materials and Methods

This study included women who underwent infertility evaluation and treatment at the Department of Obstetrics and Gynecology, University of Yamanashi, between January 2021 and May 2025. The study protocol was approved by the Ethics Committee of the University of Yamanashi (IRB approval number: 2584), and informed consent was obtained using an opt‐out approach.

The primary cohort consisted of all women who underwent conventional infertility treatment, which was defined as timed intercourse guidance and intrauterine insemination. Patients with uterine morphological abnormalities other than arcuate uterus or those in whom uterine morphology could not be evaluated by 3D‐TVUS due to imaging artifacts were excluded from the analysis. A subgroup analysis was conducted among patients who underwent assisted reproductive technology (ART) treatment. This ART subgroup consisted exclusively of patients from the primary cohort who did not achieve pregnancy through conventional infertility treatment and subsequently proceeded to ART. All ART procedures were performed within the same institution under standardized protocols, and good‐quality blastocysts graded ≥ BB according to the Gardner classification were used for all embryo transfers.

**FIGURE 1:**
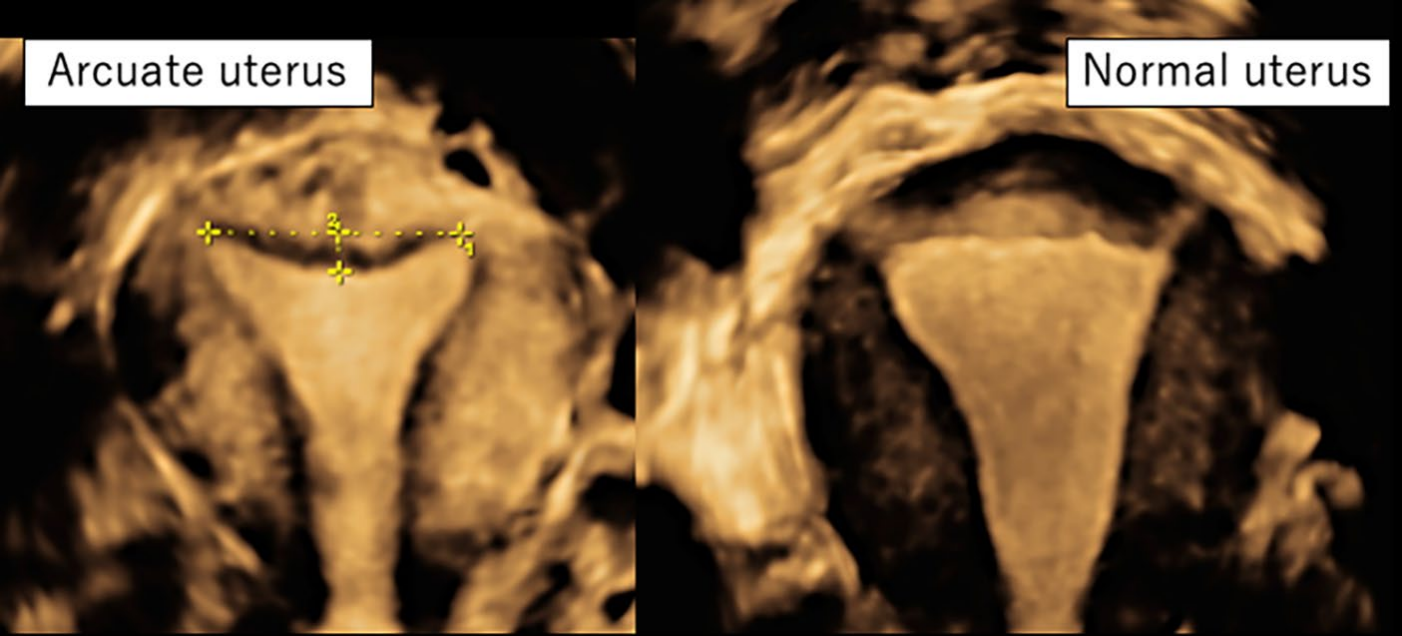
Classification of Arcuate uterus and Normal uterus based on 3D vaginal ultrasonography. Three‐dimensional ultrasound of an arcuate uterus showed a uterine cavity that was concave and a broad focal thickening of the myometrium < 1 cm in length from the inter‐osial line. Normal uterus showed that the uterine cavity was straight or convex.

All patients underwent uterine morphological assessment using 3D‐TVUS (Voluson P8, GE HealthCare, Chicago, IL, USA) during the ovulatory phase, approximately 14 days before the onset of menstruation. The midline coronal plane of the uterus was obtained by reconstructing a volume dataset acquired in the midsagittal plane. The inter‐cornual line was defined as the line connecting both tubal ostia. Uterine morphology was evaluated using a standardized protocol, including a probe frequency of 5–9 MHz and default gain settings provided for gynecologic 3D acquisition. Uterine morphology was classified based on the 2021 ASRM classification, with reference to the criteria proposed by Ghi et al. and the CUME consensus [3, 14, 16]. Classification was performed according to the following measurement criteria. An “indentation depth” of less than 10 mm was defined as the distance from the inter‐cornual line (connecting both tubal ostia) to the apex of the indentation. A “fundal angle” of ≥ 90° was defined as the angle formed between the apex of the indentation and the line connecting both tubal ostia. Cases meeting both of these criteria were classified as having an arcuate uterus. An indentation depth of ≥ 10 mm or a fundal angle of < 90° was classified as a septate uterus. Cases without an indentation, in which the uterine cavity contour was straight or convex at the fundal level, were classified as a normal uterus. Representative images are shown in Figure 1. Borderline cases—such as those with an indentation depth close to 10 mm, a fundal angle near 90°, or those with a nearly straight indentation—were reevaluated twice by the primary reviewer, and the final classification was determined strictly according to the predefined criteria to minimize misclassification.

To ensure the objectivity and reliability of uterine morphology classification, the first author (T.Y.) reevaluated a randomly selected subset of 50 3D‐TVUS datasets after an interval of at least 4 weeks. The intra‐observer reproducibility of this classification (normal vs. arcuate) was quantitatively assessed using the kappa statistic. In addition, an independent co‐author (D.M.) with expertise in 3D‐TVUS performed a second reading of another randomly selected set of 50 stored 3D datasets to assess inter‐reader reproducibility.

The primary outcome was live birth in women with arcuate and normal uterine morphology. Live birth was defined as the delivery of a live infant at ≥ 22 weeks of gestation resulting from any infertility treatment performed during the follow‐up period. The live birth rate was calculated on a per‐patient basis, rather than per oocyte retrieval or embryo transfer cycle. Secondary outcomes included the prevalence of arcuate uterus in the primary cohort, the frequency of arcuate uterus among women with unexplained infertility, and comparisons of patient characteristics and infertility etiologies between the arcuate and normal uterus groups. Similar comparisons were also performed in the ART subgroup.

Continuous variables were expressed as mean ± standard deviation (SD), and categorical variables were presented as absolute numbers (*n*) and percentages (%). Comparisons of continuous variables were performed using Student's *t*‐test, and comparisons of categorical variables were conducted using the chi‐square test or Fisher's exact test, as appropriate. To assess the independent association between arcuate uterus and live birth, a multivariable logistic regression model was used. Because the number of live birth events was limited, the number of covariates in the model was restricted to avoid overfitting. Candidate variables were selected based on clinical relevance and observed baseline imbalances, while considering variance inflation factors (VIF) to prevent multicollinearity. Variables with a VIF greater than 5 were considered for exclusion from the model. The results were presented as crude odds ratios (cOR) or adjusted odds ratios (aOR) with 95% confidence intervals (CI). As the proportion of missing data for each variable was less than 5%, complete case analysis was adopted. The overall model fit was evaluated using the Hosmer–Lemeshow goodness‐of‐fit test. A *p*‐value of < 0.05 was considered statistically significant. All statistical analyses were performed using JMP software (version 18.2.0; SAS Institute Inc., Cary, NC, USA).

## 3 Results

**FIGURE 2:**
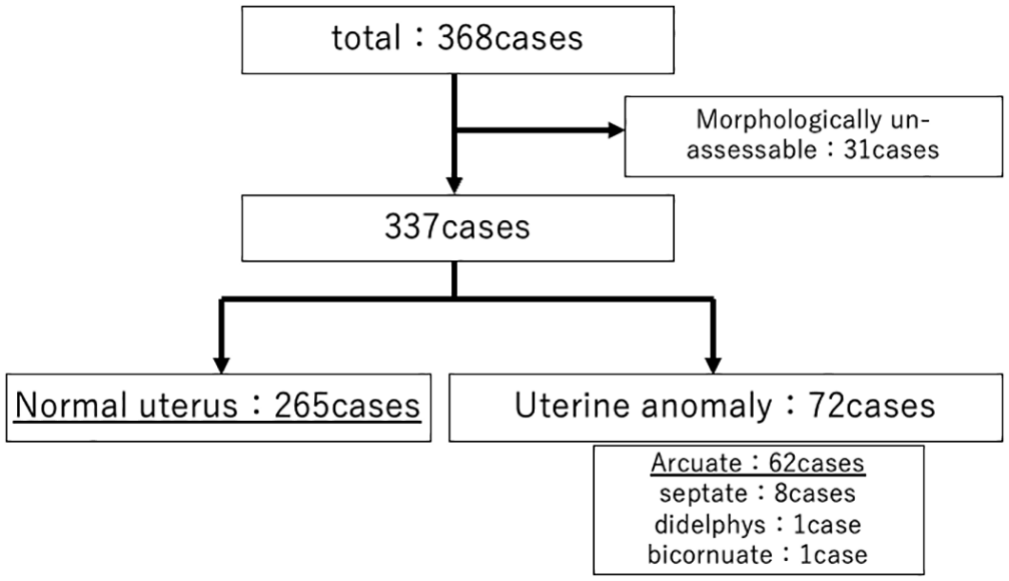
Flow diagram of study subjects in non‐ART. Morphologically unassessable cases caused by organic disorders of the uterus.

Of the 368 infertile patients enrolled during the study period, uterine morphology could be evaluated in 337. An additional 10 patients diagnosed with uterine anomalies other than arcuate uterus were excluded from the analysis. Consequently, the final primary cohort (*n* = 327) consisted of 265 patients (81%) with normal uterine morphology and 62 patients (19%) with arcuate uterus. Details are shown in Figure 2.

To ensure the objectivity and reliability of uterine morphology classification, a reevaluation was performed on a randomly selected subset of 50 cases using the stored 3D‐TVUS image data. The intra‐observer reproducibility for classifying arcuate versus normal uterus was *κ* = 0.94 (95% CI, 0.83–1.00), confirming a substantial level of diagnostic concordance. In addition, inter‐reader reproducibility was assessed using another randomly selected subset of 50 3D datasets independently reviewed by a second examiner. The inter‐reader agreement was also excellent (*κ* = 0.93, 95% CI 0.79–1.00), further supporting the robustness of the morphological classification.

Table 1 shows the comparison of patient characteristics and causes of infertility between the arcuate uterus group (*n* = 62) and the normal uterus group (*n* = 265) in the primary cohort (*n* = 327). The prevalence of unexplained infertility was significantly higher in women with arcuate uterus than in those with normal uterine morphology (47% (29/62) vs. 24% (63/265), *p* = 0.001). Moreover, compared with the normal uterus group, women with an arcuate uterus had a significantly higher body mass index (BMI) (23.1 ± 4.5 vs. 21.9 ± 3.7 kg/m^2^, *p* = 0.02) and a higher prevalence of polycystic ovary syndrome (PCOS) (13% (8/62) vs. 4.2% (11/265), *p* = 0.001). In contrast, infertility due to female factors was significantly less frequent in the arcuate uterus group (37% (23/62) vs. 54% (142/265), *p* = 0.02). No significant differences were observed between the two groups for other factors.

**TABLE 1:** Comparison of patient characteristics and causes of infertility between the arcuate uterus group and the normal uterus group in non‐ART cases.

	Arcuate uterus (*n* = 62)	Normal uterus (*n* = 265)	*p*
Age	35.7 ± 4.4	35.8 ± 4.8	0.79
BMI (kg/m^2^)	23.1 ± 4.5	21.9 ± 3.7	0.02*
AMH (ng/mL)	3.1 ± 2.8	2.8 ± 4.8	0.50
Live birth	11 (17%)	37 (14%)	0.47
History of miscarriage	12 (19%)	82 (31%)	0.07
Myoma	13 (21%)	47 (18%)	0.56
Adenomyosis	4 (6.5%)	15 (5.7%)	0.82
Endometriosis	7 (11%)	51 (19%)	0.14
Endometrial polyp	10 (16%)	24 (9.1%)	0.10
PCOS	8 (13%)	11 (4.2%)	0.001*
Female factor	23 (37%)	142 (54%)	0.02*
Male factor	10 (16%)	36 (14%)	0.60
Unexplained	29 (47%)	63 (24%)	0.001*

*Note:* The arcuate uterus (*n* = 62) and the normal uterus (*n* = 265) are shown. Data are presented as mean ± SD and *n* (%). Moreover, *p* values are assessed using the chi‐squared test, the Mann–Whitney *U* test and the Fisher's exact test (*p < 0.05).

Abbreviations: AMH, anti‐Mullerian hormone; ART, assisted reproductive technology; BMI, body mass index; PCOS, polycystic ovary syndrome; SD, standard deviation.

Among all patients in the primary cohort (*n* = 327), 92 (28%) were diagnosed with unexplained infertility. In the unexplained infertility group (*n* = 92), 29 women (32%) had an arcuate uterus, whereas in the group with identifiable infertility factors (*n* = 235), 33 women (14%) had an arcuate uterus. The odds of having an arcuate uterus were significantly higher among women with unexplained infertility (cOR 2.82, 95% CI 1.59–5.00, *p* = 0.001), suggesting that universal 3D‐TVUS screening may be useful for identifying a specific subgroup within unexplained infertility.

Regarding the primary outcome—the effect of arcuate uterus on live birth in the primary cohort (*n* = 327)—the live birth rate throughout all treatment cycles was 17% (11/62) in the arcuate uterus group and 14% (37/265) in the normal uterus group, with no significant difference between the two (cOR 1.33, 95% CI 0.64–2.78, *p* = 0.43). In a multivariable logistic regression analysis adjusted for arcuate uterus, BMI, anti‐Müllerian hormone (AMH), ovulatory disorder, and male factor infertility, the presence of an arcuate uterus was not independently associated with live birth (aOR 1.3, 95% CI 0.2–5.9, *p* = 0.82) (Table 2).

**TABLE 2:** Univariable and multivariable logistic regression for predictors of live birth in the non‐ART cohort, comparing women with arcuate and normal uterus.

	Live birth (*n* = 48)	Nonlive birth (*n* = 279)	*p*	cOR (95% CI)	aOR (95% CI)	*p*
Arcuate uterus	11 (23%)	51 (18%)	0.46	1.3 (0.6–2.8)	1.3 (0.2–5.9)	0.82
Male factors	5 (10%)	41 (15%)	0.43	0.7 (0.3–1.8)	0.7 (0.2–1.7)	0.41
Ovulatory disorders	8 (17%)	37 (13%)	0.53	1.3 (0.6–3.0)	1.3 (0.5–2.9)	0.54
BMI (kg/m^2^)	22.5 ± 4.5	22.1 ± 3.8	0.69	1.0 (0.9–1.1)	0.9 (0.7–1.1)	0.31
AMH (ng/mL)	1.6 ± 1.2	2.9 ± 2.7	0.11	0.7 (0.4–1.1)	0.7 (0.4–1.1)	0.15

*Note:* Data are shown as mean ± SD or *n* (%). Group‐comparison *p* values are from the *χ* ^2^ test, Fisher's exact test, or the Mann–Whitney *U* test, as appropriate. cORs are obtained from univariable logistic regression comparing arcuate versus normal uterus and other covariates. aORs are obtained from a multivariable logistic regression model including arcuate uterus, BMI, AMH, male factor infertility, and ovulatory disorders. For continuous variables, unit odds ratios are reported: BMI per 1 kg/m^2^ and AMH per 1 ng/mL.

Abbreviations: AMH, anti‐Müllerian hormone; aOR, adjusted odds ratio; ART, assisted reproductive technology; BMI, body mass index; CI, confidence interval; cOR, crude odds ratio; PCOS, polycystic ovary syndrome; SD, standard deviation.

A similar analysis was performed in the ART subgroup (*n* = 233), which included 41 women with an arcuate uterus and 192 with normal uterine morphology. Table 3 shows the comparison of patient characteristics and causes of infertility between the two groups. The prevalence of unexplained infertility was significantly higher among women with an arcuate uterus (46% (19/41) vs. 28% (53/192), *p* = 0.02). Additionally, the arcuate uterus group had a significantly higher BMI (22.4 ± 5.0 vs. 21.3 ± 4.8 kg/m^2^, *p* = 0.03) and a higher incidence of endometrial polyps (24% (10/41) vs. 11% (21/192), *p* = 0.02) compared with the normal uterus group. No significant differences were observed between the two groups for other factors.

**TABLE 3:** Comparison of patient characteristics and causes of infertility between the arcuate uterus group and the normal uterus group in ART.

	Arcuate uterus (*n* = 41)	Normal uterus (*n* = 192)	*p*
Age	37.0 ± 4.6	36.4 ± 4.7	0.49
BMI (kg/m^2^)	22.4 ± 5.0	21.3 ± 4.8	0.03*
AMH (ng/mL)	3.1 ± 2.9	2.9 ± 2.7	0.73
Live birth	26 (65%)	119 (63%)	0.84
History of miscarriage	11 (27%)	64 (33%)	0.42
Myoma	8 (20%)	33 (17%)	0.72
Adenomyosis	3 (7.3%)	13 (6.8%)	0.90
Endometriosis	4 (9.8%)	43 (22%)	0.07
Endometrial polyp	10 (24%)	21 (11%)	0.02*
PCOS	4 (9.8%)	7 (3.7%)	0.09
Female factor	17 (41%)	112 (58%)	0.05
Male factor	6 (15%)	31 (16%)	0.80
Unexplained	19 (46%)	53 (28%)	0.02*

*Note:* The arcuate uterus (*n* = 41) and the normal uterus (*n* = 192) are shown. Data are presented as mean ± SD and *n* (%). Moreover, *p*‐values are assessed using the chi‐squared test, the Mann–Whitney *U* test and the Fisher's exact test (*p < 0.05).

Abbreviations: AMH, anti‐Mullerian hormone; ART, assisted reproductive technology; BMI, body mass index; PCOS, polycystic ovary syndrome; SD, standard deviation.

Among all patients in the ART subgroup (*n* = 233), 72 (31%) were diagnosed with unexplained infertility. In the unexplained infertility group (*n* = 72), 19 women (26%) had an arcuate uterus, whereas in the group with identifiable infertility factors (*n* = 163), 22 women (13%) had an arcuate uterus. The odds of having an arcuate uterus were significantly higher among women with unexplained infertility (cOR 2.30, 95% CI 1.15–4.58, *p* = 0.02), suggesting that universal 3D‐TVUS screening may be useful for identifying a specific subgroup within unexplained infertility.

Regarding the impact of arcuate uterus on live birth in the ART subgroup (*n* = 233), the live birth rate across all treatment cycles was 65% (26/41) in the arcuate uterus group and 63% (119/192) in the normal uterus group, with no significant difference between the two (cOR 1.06, 95% CI 0.53–2.14, *p* = 0.99). In a multivariable logistic regression analysis adjusted for arcuate uterus, body mass index (BMI), anti‐Müllerian hormone (AMH), ovulatory disorder, and male factor infertility, the presence of an arcuate uterus was not independently associated with live birth (aOR 1.1, 95% CI 0.5–2.4, *p* = 0.82) (Table 4).

**TABLE 4:** Univariable and multivariable logistic regression for predictors of live birth in the ART cohort, comparing women with arcuate and normal uterus.

	Live birth (*n* = 145)	Nonlive birth (*n* = 88)	*p*	cOR (95% CI)	aOR (95% CI)	*p*
Arcuate uterus	26 (18%)	14 (16%)	0.86	1.1 (0.5–2.2)	1.1 (0.5–2.4)	0.82
Male factors	26 (18%)	10 (11%)	0.26	1.6 (0.7–3.4)	1.1 (0.5–2.7)	0.76
Ovulatory disorders	17 (12%)	8 (9.61%)	0.64	1.2 (0.5–3.0)	1.2 (0.4–3.1)	0.71
BMI (kg/m^2^)	21.4 ± 3.7	22.7 ± 4.2	0.03*	0.9 (0.8–0.9)	0.9 (0.8–0.9)	0.02*
AMH (ng/mL)	3.3 ± 2.8	2.1 ± 2.4	< 0.001*	1.2 (1.1–1.4)	1.2 (1.1–1.4)	< 0.001*

*Note:* Data are shown as mean ± SD or *n* (%). Group‐comparison *p* values are from the *χ* ^2^ test, Fisher's exact test, or the Mann–Whitney *U* test, as appropriate. cORs are obtained from univariable logistic regression comparing arcuate versus normal uterus and other covariates. aORs are obtained from a multivariable logistic regression model including arcuate uterus, BMI, AMH, male factor infertility, and ovulatory disorders. For continuous variables, unit odds ratios are reported: BMI per 1 kg/m^2^ and AMH per 1 ng/mL. (*p* < 0.05*).

Abbreviations: AMH, anti‐Müllerian hormone; aOR, adjusted odds ratio; ART, assisted reproductive technology; BMI, body mass index; CI, confidence interval; cOR, crude odds ratio; PCOS, polycystic ovary syndrome; SD, standard deviation.

## 4 Discussion

In this study, uterine morphology in infertile women was systematically evaluated using 3D‐TVUS to investigate the prevalence and clinical significance of arcuate uterus. As a result, arcuate uterus was identified in approximately 19% (62/327) of all infertility cases, with a notably higher frequency among women with unexplained infertility. In contrast, no significant difference was observed in live birth rates, suggesting that the presence of an arcuate uterus is unlikely to have a direct impact on infertility treatment outcomes.

According to the 2021 ASRM classification [14], the arcuate uterus is defined as a “transitional form between normal and septate uterus,” yet its clinical significance remains a matter of ongoing debate. Previous studies have reported conflicting findings—some suggesting that an arcuate uterus may increase the risk of infertility or miscarriage, while others have found no adverse effect on pregnancy outcomes—thus, no consensus has been reached [6, 8, 9, 17, 18, 19].

In the present study, a notably high prevalence of arcuate uterus was observed among women with unexplained infertility. Arcuate uterus was identified in 32% (29/92) of women with unexplained infertility, which was significantly higher compared with those with explained infertility (cOR 2.82, 95% CI 1.59–5.00, *p* = 0.001). However, no significant difference was found in live birth rates (aOR 1.3, 95% CI 0.2–5.9, *p* = 0.82). Similarly, in the ART subgroup, arcuate uterus was significantly more frequent among women with unexplained infertility.

These findings suggest the hypothesis that subtle morphological variations at the uterine fundus could be associated with early reproductive processes such as fertilization or implantation. However, once conception occurs—particularly under assisted reproductive conditions—such anatomical variations might have only a limited influence on pregnancy maintenance or live birth. Possible underlying mechanisms have been hypothesized, including altered endometrial surface morphology or vascular distribution at the fundal region caused by mild indentation, and localized differences in endometrial receptivity [4, 13, 20, 21]. However, as uterine blood flow and endometrial function were not directly assessed in this study, further research is warranted to elucidate these physiological associations.

The strength of this study lies in its comprehensive evaluation of uterine morphology using 3D‐TVUS in all infertility cases, with classification based on clearly defined and objective criteria. In addition, the high intra‐observer reproducibility (*κ* = 0.94) supports the robustness and reliability of the classification, further enhancing the credibility of this study.

On the other hand, this study has several limitations. First, it was a retrospective, single‐center study, and therefore the possibility of selection bias or unmeasured confounding factors cannot be completely excluded. Second, the present analysis focused on live birth as the primary outcome, and detailed evaluations of pregnancy course, such as miscarriage or preterm birth, were not performed. Third, functional parameters such as endometrial blood flow and hormonal dynamics were not assessed; thus, the pathophysiological association between arcuate uterus and unexplained infertility remains speculative. Fourth, because the number of live birth events—particularly in the non‐ART subgroup—was limited, the statistical power to detect small differences was insufficient. Therefore, the lack of a statistically significant association should be interpreted with caution, as a modest effect cannot be completely excluded. Although no statistically significant association was found, the wide confidence intervals indicate a degree of uncertainty in the estimates. Thus, the results should not be interpreted as conclusive evidence of no effect.

Based on these findings, arcuate uterus was identified in approximately one in five women with infertility and was more frequently observed among those with unexplained infertility. However, it did not exert an independent effect on infertility treatment outcomes or live birth rates. Morphological assessment using 3D‐TVUS may be useful for understanding the underlying pathophysiology of unexplained infertility, but further prospective multicenter studies are warranted to determine whether therapeutic intervention is necessary.

## Funding

The authors have nothing to report.

## Ethics Statement

The ethics committee of Yamanashi University (ref approval No. 2584) authorized the research. All procedures were carried out following relevant guidelines and regulations and with the 1964 Helsinki Declaration and its later amendments or comparable ethical standards.

## Conflicts of Interest

The authors declare no conflicts of interest.

## Data Availability

The data that support the findings of this study are available on request from the corresponding author. The data are not publicly available due to their containing information that could compromise the privacy of research participants.
